# Multiple ossified spinal meningiomas in the thoracic spine: A case report and literature review

**DOI:** 10.3389/fsurg.2022.965815

**Published:** 2022-10-04

**Authors:** Chunke Dong, Yi Liu, Yuting Zhu, Hongyu Wei, Yuzhuo Ma

**Affiliations:** ^1^ Beijing Hospital of Traditional Chinese Medicine, Capital Medical University, Beijing, China; ^2^ Department of Medical Rehabilitation, Yan Liang Hospital District of Xi’an Honghui Hospital, Xi’an, China; ^3^ Section III of Internal Medicine Department, Tongzhou District Hospital of Integrated Traditional Chinese Medicine and Western Medicine, Beijing, China; ^4^ Department of Spine Surgery, China-Japan Friendship Hospital, Beijing, China; ^5^ Department of Orthopedics, Honghui Hospital, Xi'an Jiaotong University, Xi’an, China

**Keywords:** multiple meningiomas, spinal meningioma, ossification, calcification, surgery

## Abstract

**Background:**

Ossified spinal meningioma (OSM) is a rare form of a spinal tumor. The surgical strategies and pathologic findings related to OSM have been investigated in recent years. However, multiple OSMs are rarely reported. Here, we intend to present a rare case of multiple OSMs and review the relevant published literature.

**Case Presentation:**

A 76-year-old woman experienced a progressive sensorimotor disturbance in her bilateral lower limbs for the past 2 years. She complained of inability to walk, urinary incontinence, and chronic constipation when referred to our hospital. A neurological examination revealed a diminished sensation below the bilateral T7, and her neurological status was Nurick Grade 6. Magnetic resonance imaging (MRI) revealed multiple intradural-extramedullary neoplasms at the T7–T11 level. Computed tomography (CT) scans showed five high-density masses of varying sizes in the spinal canal at the T7–T12 level. The patient underwent tumor resection through T7–T11 laminectomy. A histopathological examination revealed multiple OSMs.

**Conclusion:**

We reported a rare case of multiple OSMs in an elderly patient. After one-stage complete resection, the patient recovered with satisfactory curative effect. Although elderly patients will face various postoperative complications due to their poor physical condition, we still recommend one-stage complete resection of multiple OSMs to reduce recurrence.

## Introduction

Meningioma is the second most common of all primary spine tumors, accounting for 25%–45% (1). Multiple meningiomas usually occur inside the cranium and occasionally in both the skull and the spinal canal (2). While intracranial meningiomatosis presents as solid, cystic, or solid-cystic lesions, ranging between WHO Grade I and III, multiple spinal meningiomas are usually homogeneously solid WHO Grade I lesions (3). Ossified spinal meningioma (OSM), a rare form of meningioma, accounts for 0.7%–5.5% of all spinal meningiomas (4–6). However, only one case of multiple OSMs has been reported to date (7). Here, we present the first exclusive case of multiple OSMs with more than two independently situated meningiomas in a 76-year-old woman and a review of the literature.

## Case presentation

### Medical history

**Figure 1:**
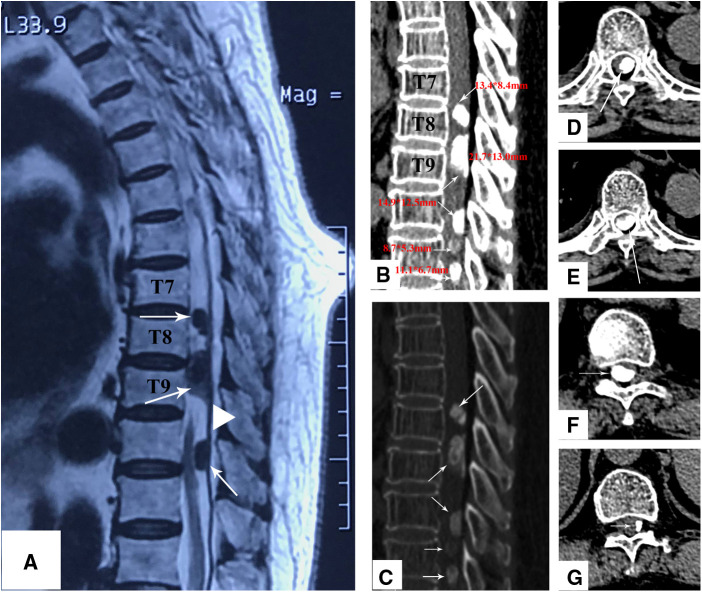
(**A**) Sagittal T2-weighted MRI shows three large lesions at the T7–T11 level with low signal intensity. The lesions shown with a dural tail sign. (**B,C**) The sagittal CT scan shows that there are five different sizes of high-density masses at the T7–T8 (**D**), T8–T9 (**E**), T10–T11 (**F**), and T11–T12 (**G**), levels, and all of them are located at the posterior of the spinal canal.

A 76-year-old woman reported a history of progressive sensorimotor disturbance in both lower limbs for the past 2 years. Initially, the patient’s condition was misdiagnosed as “lumbar disc herniation” in the local hospital, and she received conservative treatment. However, her symptoms did not improve and became more serious. When referred to our hospital, she complained of inability to walk and bladder and bowel control loss. A neurological examination revealed a diminished sensation below the T7 level. The strength of both lower limb muscles was 1/5. Her neurological status was diagnosed as Nurick Grade 6. Magnetic resonance imaging (MRI) revealed multiple intradural extramedullary tumors at the T7–T11 level compressing the spinal cord (Figure 1A). Computed tomography (CT) scans showed five high-density masses of varying sizes in the spinal canal at the T7–T12 level, and the dural sac was significantly compressed (Figures 1B,C). Three large high-density masses (13.4 mm*8.4 mm, 21.7 mm*13.0 mm, and 14.9 mm*12.5 mm) were observed in the T7–T9 segment, located in the dorsal left rear spinal cord with clear boundaries and ossification signals (Figures 1D–F). The other two high-density masses (8.7 mm*5.3 mm and 11.1 mm*6.73 mm) of different sizes were found in the T10–T12 segment.

### Surgical management

The patient underwent tumor resection through T7–T11 laminectomy. The tumors occupied >90% of the transverse diameter of the spinal canal and were entirely ossified. As the vertebral fenestration was too small, it was challenging to remove the tumors. Hence, the bilateral facet joints were removed to enlarge the window. To maintain the stability of the spine, we performed T7–T12 long-segment pedicle screw fixation and posterolateral bone graft fusion simultaneously. Bilateral facetectomy provides advantages over simple laminectomy or laminoplasty in terms of width of the operative corridor and long-term preservation of the spinal alignment. As the dura mater had severely adhered, a fine right-angled hook was used to dissect the neural tissue dorsally away from the neoplasms, which were then resected *en bloc*, together with parts of the dura mater and arachnoid. After a complete separation of multiple OSMs, the dura mater became normal and it was sutured. The drainage was routinely placed, and the wound was closed layer by layer.

### Histopathological outcomes

**Figure 2:**
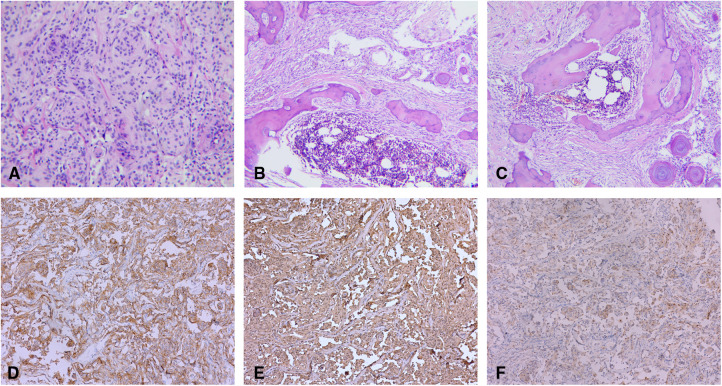
(**A**) Tumors are demonstrated to be a spinal meningioma on histology (H/E stain, original magnification ×100). (**B,C**) Psammoma bodies around the tumor cells. Mature bone tissues around the tumor cells, including trabecular bone, as well as bone marrow with hematopoiesis (H/E stain, original magnification ×40). Immunohistochemically, the neoplastic meningothelial cells are immunoreactive for (**D**) epithelial membrane antigen (EMA) (×10), (**E**) vimentin (×10), and (**F**) soluble protein-100 (S-100) (×10).

**Figure 3:**
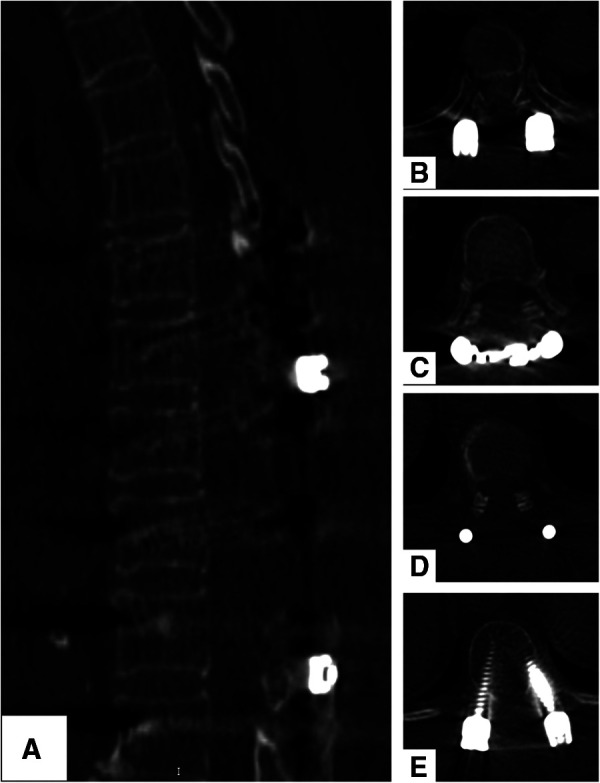
(**A–E**) CT scans show a satisfactory decompression of the spinal cord, and no recurrence spinal meningioma was found after 2 years.

The paraffin sections were stained with hematoxylin and eosin (H/E). A histopathological examination revealed a large number of psammoma bodies in the stroma with significant signs of ossification [World Health Organization (WHO) Grade I]. In addition, there were many mature bone tissues around the tumor cells, including trabecular bone, as well as bone marrow with hematopoiesis (Figures 2A–C). Immunohistochemical findings revealed EMA (+), Vim (+), P53 (−), S-100 (+), and Ki67 (approximately 5%) (Figures 3D–F).

### Postoperative and follow-up results

Postoperative neurological improvement was significant, and no complications were found. The patient was discharged 2 weeks after surgery. After the 2-year follow-up, she was able to walk without assistance. Her neurological status recovered to Nurick Grade 3. CT scans showed a total resection of the tumors, and there was no recurrence 2 years after surgery (Figures 3A–E).

## Discussion

Including our current report, 33 articles (4, 7–37) containing 43 cases of OSMs have been published as of this year, according to PubMed. (In Table 1, the search terms are ((((((ossified [Title/Abstract]) OR (osteoblastic [Title/Abstract])) OR (osseous metaplasia [Title/Abstract])) OR (psammomatous [Title/Abstract])) OR (calcified [Title/Abstract])) AND (spinal [Title/Abstract])) AND (meningioma [Title/Abstract]), and 76 potential studies are identified. We also checked the reference lists of all, including articles, to add 10 other articles.) For this condition, female predominance has been clearly noted (female, 38; male, 5), and the average age is 58.4 years, ranging from 15 to 90. Most tumors are located in the thoracic spine, except four in the cervical region and one in the lumbar region. Bone formation and hematopoiesis have been found in 7 cases, and only 2 cases of multiple OSMs have been identified, including our report.

**Table 1:** Summary of ossified meningioma cases.

Study	Age/Sex	Level	Tumor number	Symptoms	Histological characteristics
Roger (18)	16/F	T9	1	Myelopathy	Psammoma bodies, bone cells
Freidberg (29)	69/F	T1–T2	1	Myelopathy	Psammoma bodies, mature cancellous bone
Kandel et al. (30)	17/F	T8	1	Myelopathy	Meningotheliomatous, psammoma bodies, bone spicule
Niijima et al. (4)	75/F	T8–T9	1	Myelopathy	Psammoma bodies, bone spicule
Kitagawa et al. (32)	75/F	T9–T10	1	Myelopathy	Psammoma bodies, bone tissue
	60/F	T6–T8	1	Myelopathy	Psammoma bodies, bone tissue
Nakayama et al. (33)	74/F	T9	1	Myelopathy	Matured lamellar bone tissue
	45/M	C1–C3	1	Myelopathy	Matured bone tissue
Huang et al. (34)	73/F	T5	1	Myelopathy	Psammoma bodies, bone marrow
Saito et al. (31)	54/F	T11	1	NA	Metaplastic (osseous)
Naderi et al. (35)	15/M	T4	1	Myelopathy	Psammoma bodies, mature bone tissue
Liu et al. (36)	70/F	T11	1	Myelopathy	Psammoma bodies, woven bone
Hirabayashi et al. (37)	82/F	L3	1	Cauda equina syndrome	osseous
Tahir et al. (8)	40/F	T6	1	Myelopathy	Mineralized bone
Uchida et al. (7)	76/F	T8 and T12	2	Myelopathy	Psammoma bodies, mature bone
Licci et al. (9)	58/F	T6	1	Myelopathy	Psammoma bodies, lamellar bone tissue, hematopoiesis
Chotai et al. (12)	61/F	T4–T5	1	Myelopathy	Psammoma bodies, mature lamellar bone, hematopoiesis
Ju et al. (10)	61/F	T9–T10	1	Myelopathy	Heterotopic ossification
Taneoka et al. (11)	78/F	T9	1	Myelopathy	Psammoma bodies, mature bone, hematopoiesis
Yamane et al. (14)	61/F	T12	1	Myelopathy	Psammoma bodies, cancellous bone with bone marrow
Chan et al. (13)	64/F	T9-10	1	Myelopathy	Psammoma bodies, bone marrow, hematopoiesis
Alafaci et al. (15)	45/M	T2–T3	1	Myelopathy	Seven cases of osseous component in association with psammoma bodies, Two cases of immature bone trabeculae
	75/F	T3–T4	1	Myelopathy	
	86/F	T3–T4	1	Myelopathy	
	65/F	T7	1	Myelopathy	
	72/F	C7	1	Myelopathy	
	40/F	T1–T2	1	Myelopathy	
	65/F	T7–T8	1	Myelopathy	
	40/F	C7	1	Myelopathy	
	41/F	T2–T3	1	Myelopathy	
Demir et al. (17)	26/F	T9–T11	1	Myelopathy	Psammoma bodies
Cochran et al. (19)	47/F	T8	1	Radiculopathy	Psammoma bodies, bone marrow, hematopoiesis
Xia and Tian (16)	90/M	T10–T11	1	Spinal cord injury after fall	Psammoma bodies, bone trabeculae
Prakash et al. (20)	60/F	T7–T8	1	Myelopathy	Psammoma bodies, immature bony trabeculae
Sakamoto et al. (21)	57/F	C7	1	Myelopathy	Osseous core, fibrous
Murakami et al. (23)	29/F	T12	1	Back pain, leg numbness	Psammoma bodies, mature bone tissue
Taha et al. (22)	22/F	T4–T5	1	Myelopathy	Psammoma bodies, bone trabeculae
Wang et al. (24)	52/F	T4	1	Back pain	Psammoma bodies, immature trabecular bone, hematopoiesis
Xu et al. (25)	85/F	T11	1	Back pain, leg pain	Psammoma bodies
Buchanan et al. (26)	64/M	T4	1	Myelopathy	Psammoma bodies, bone formation, osseous metaplasia
Wong et al. (27)	75/F	T10–T11	1	Myelopathy	Psammoma bodies, immature trabeculae bone
Thakur et al. (28)	74/F	T8	1	Tingling paresthesia	Psammoma bodies, bony hard-tissue fragments
Present case	76/F	T7–T12	5	Myelopathy	Psammoma bodies, trabecular bone, hematopoiesis

Multiple meningiomas are defined as more than two independently situated meningiomas arising simultaneously or sequentially (2). The pathogenesis of multiple meningiomas remains elusive. In fact, at present, two kinds of hypotheses are proposed by the relevant literature: one supports the theory of monoclonal spreading and the other suggests that of clonally unrelated onset for lesions arising sequentially from two clearly distinct spinal regions (38). A genomic profiling study reveals that multiple meningiomas can be of both mono- and multiclonal origin. Even monoclonal multiple meningiomas can acquire intertumor heterogeneity through branched evolution resulting in pathology, and the landscape of one tumor may not be representative of the others. Thus, multiple meningiomas should be tailored individually if feasible (39).

The concept of ossification of meningioma should be distinguished from calcification. Calcification is more a radiologic description than a histopathological diagnosis and is commonly seen in psammomatous meningioma. Based on the classification by the World Health Organization (WHO), ossification in meningioma is histologically classified as a subtype of metaplastic meningioma and is characterized by the expression of mesenchymal components (40). The occurrence of OSM also remains unclear. One hypothesis is that ossification results from the repeated accumulation of hydroxyapatite crystals in psammoma bodies (41). However, some reports (4, 8, 10, 15, 21) have occasionally found that ossification can occur without psammoma bodies, which may not support the hypothesis. Hence, most studies prefer to believe that ossification is secondary to the metaplasia of arachnoid cells and interstitial cells, which induce the synergistic effect of osteoblast, fibroblast, and angiogenesis components in bone tissue formation (4, 8, 10, 23). Up to now, seven studies have mentioned the formation of hematopoietic tissue in OSM. This unexpected finding seems to confirm the mesenchymal potential of meningiomatous cells, which may undergo bone metaplasia (9, 11–13, 19, 24).

Although OSM was previously reported to grow very slowly and experience a long asymptomatic period, the symptoms may appear at an early stage in intraspinal tumors compared with intracranial tumors due to a smaller space in the intraspinal canal (15). As most commonly in WHO Grade I, multiple spinal meningiomas can be considered benign lesions with a good prognosis, especially if the lesion and affected dura are macroscopically complete removed (Simpson grade I) (42). Mirimanoff et al. (43) found that the incidence rates of secondary operation after a total resection for follow-up periods of 5, 10, and 15 years were 6%, 15%, and 20%, respectively. In contrast, after a subtotal resection, the probability was 25%, 44%, and 84%, respectively.

Compared with the common meningiomas, OSM closely adheres to the dura and arachnoid (7). If the tumor and the dura mater are not separated, they could be removed together and repaired with an artificial dura mater covered with gelatin sponge. Ruggeri et al. (44) proposed that a poor surgical outcome in patients with ossified tumors results from a more “invasive” surgical removal of an ossified mass: an internal debulking is not feasible for a hard tumor. In our case, the ossified tumors occupied the entire transverse diameter of the spinal canal and could be approached more safely with laminectomy by removing the bilateral facet joints from T7 to T12 instead of with hemilaminectomy: it offers a wider operative field for a better manipulation of the neural tissue, especially the thoracic spinal cord, which is more susceptible to damage than the cervical spinal cord and lumbar spinal roots. Therefore, we performed a T7–T12 long-segment pedicle screw fixation and posterolateral bone graft fusion to prevent the occurrence of iatrogenic kyphosis and maintain the stability of the spine. After a 2-year follow-up, no complications such as internal fixation failure and kyphosis occurred. The use of intraoperative ultrasound represents a valuable surgical aid for real-time neuronavigation, allowing the operating team to evaluate the decompression of the spinal cord and rule out subdural blood clots at the time of dural closure/reconstruction (45). For spinal intradural extremedullary lesions, the sensitivity, specificity, and positive and negative predicted values of intraoperative neurophysiological monitoring (IONM) are reported to be 75, 100, 100, and 97%, respectively. This indicates that IONM predicts neurological deficits with high accuracy, although its role in preventing new neurological deficits in spinal meningiomas has yet to be proved (46). The current trends in the use of adjuvant radiotherapy and radiosurgery for spinal meningiomas have increased; nonetheless, this has not led to a significant increase in overall survival rates (47). Larger tumor size and borderline or malignant behavior are reported to be associated with increased radiation use; the introduction of multimodal adjuvant technologies such as radioenhancers has yet to provide evidence for superior outcomes (48). To sum up, surgical en bloc resection of OSM is difficult, especially in multiple OSMs, but it is still the best treatment.

Although ossified meningioma could be identified in a plain radiograph, CT and MRI are complementary methods of diagnosing a calcified spinal meningioma, especially in cases replaced entirely by calcification (49). On MR images, the signal intensities of calcifications within the masses were variable, and the extent of signal intensities suggesting calcification did not concur with that of calcified foci within a mass, as seen on CT images (49).

## Conclusion

In the current report, we present a rare case of multiple OSMs in the thoracic spine. Although the mechanism of occurrence is not clear, a total resection is generally required, and a satisfying prognosis and a low recurrence rate can be expected. The surgical strategy for OSMs differs from that for other meningiomas. We suggest that preoperative CT be used to accurately locate ossification. Intraoperative use of a wide surgical corridor with total laminectomy, combined with bilateral facet joint resection, identification of upper and lower poles, and early CSF drainage, is helpful in decreasing neural retraction.

## Data Availability

The original contributions presented in the study are included in the article/Supplementary Material, further inquiries can be directed to the corresponding author.
